# Propagation-based phase-contrast x-ray tomography of cochlea using a compact synchrotron source

**DOI:** 10.1038/s41598-018-23144-5

**Published:** 2018-03-21

**Authors:** Mareike Töpperwien, Regine Gradl, Daniel Keppeler, Malte Vassholz, Alexander Meyer, Roland Hessler, Klaus Achterhold, Bernhard Gleich, Martin Dierolf, Franz Pfeiffer, Tobias Moser, Tim Salditt

**Affiliations:** 1 Institute for X-Ray Physics, University of Göttingen, Göttingen, Germany; 2 Center for Nanoscopy and Molecular Physiology of the Brain, Göttingen, Germany; 3 Chair of Biomedical Physics, Department of Physics, Technical University of Munich, Garching, Germany; 4 Institute for Advanced Study, Technical University of Munich, Garching, Germany; 5 Munich School of BioEngineering, Technical University of Munich, Garching, Germany; 6 Institute for Auditory Neuroscience and InnerEarLab, University Medical Center Göttingen, Göttingen, Germany; 7 InnerEarLab, Department of Otolaryngology, University Medical Center Göttingen, Göttingen, Germany; 8 MED-EL Company, Innsbruck, Austria; 9 Department of Diagnostic and Interventional Radiology, Klinikum rechts der Isar, Technical University of Munich, München, Germany; 10 Bernstein Focus for Neurotechnology, University of Göttingen, Göttingen, Germany

## Abstract

We demonstrate that phase retrieval and tomographic imaging at the organ level of small animals can be advantageously carried out using the monochromatic radiation emitted by a compact x-ray light source, without further optical elements apart from source and detector. This approach allows to carry out microtomography experiments which - due to the large performance gap with respect to conventional laboratory instruments - so far were usually limited to synchrotron sources. We demonstrate the potential by mapping the functional soft tissue within the guinea pig and marmoset cochlea, including in the latter case an electrical cochlear implant. We show how 3d microanatomical studies without dissection or microscopic imaging can enhance future research on cochlear implants.

## Introduction

The three-dimensional (3d) structure of tissues and organs enables their physiological functions, while pathological states are often accompanied by corresponding structural alterations. At the same time, many therapeutic approaches and in particular implants also intervene in an intrinsically 3d manner with tissues and organs. The cochlea as the auditory end-organ in the inner ear is a perfect example. Quantitative shape and volume information about the complex cochlea morphology is required to indicate correct positioning of the cochlear implant (CI), including soft tissues such as the basilar membrane, Rosenthal’s canal and the auditory nerve, even though surrounded by bone^1^. Such structural data precedes the understanding of malformations caused by genetic defects, to improve novel therapeutic approaches to hearing disorders and to optimize the design of CIs^2,3^.

More generally, validation of novel diagnostic and therapeutic approaches targeting many tissues and organs should include 3d imaging at suitable resolution and contrast. Conventional histology is based on two-dimensional (2d) sections, imaged by optical microscopy. Compared to 3d imaging it faces several major deficits and restrictions. Apart from possible slicing or staining artifacts it is extremely tedious and time consuming to record the entire organ or large field of views (FOVs), making it almost impossible to cover the complete 3d tissue architecture of specimens, even at moderate resolution. Contrarily, high resolution phase-contrast x-ray tomography is capable to assess the native 3d structure of tissues with selectable FOV, for example in the range of several mm, and voxel sizes in the range of several μm. By zooming into selected regions of interest, sub-micron resolution and imaging of single cells with sub-cellular resolution can be achieved in thick tissue slices^4^.

While cellular imaging by optical microscopy has thrived over the last two decades, high resolution 3d imaging of tissues and organs by micro x-ray tomography (μ-CT) is, however, still far from being routinely available. Notably, the implementation of μ-CT is largely hampered by two factors: (i) the limited brilliance of laboratory x-ray sources, and (ii) the limited contrast of weakly or non-absorbing soft tissues. Synchrotron radiation (SR), where the illumination is sufficiently monochromatic and coherent, has enabled high image quality for tissues by phase-contrast tomography, exploiting contrast formation by free wave propagation between sample and detector^5–11^. Conversely, compact laboratory instrumentation largely lacks such abilities, even if the possibility to partially translate capabilities of SR phase-contrast tomography to the laboratory is now emerging, for example based on liquid-metal-jet anodes^1,12–14^.

An important and promising role in this respect is the advent of tabletop synchrotron sources such as the Munich Compact Light Source (MuCLS) which is based on the interaction of accelerated electrons and laser photons (inverse Compton effect)^15^. It can produce nearly monochromatic x-ray photons^16^ in a continuously tunable energy spectrum with partial spatial coherence which allows for high quality phase-contrast imaging^17^ without the drawbacks created by the typical polychromatic illumination of laboratory x-ray sources^16,18^.

Here we present 3d imaging at the small animal organ level, using the MuCLS^19^. We demonstrate the potential in a proof-of-concept study, imaging two cochleae of guinea pig and marmoset, including an electrical CI, at a resolution in the range of 10 m. The higher and tunable photon energy and in particular the narrow bandpass allows in principle for more quantitative reconstruction values (grey levels) than possible with conventional laboratory microfocus x-ray sources, and in particular the liquid-metal-jet source used in earlier studies of mouse cochlea^1^. We expect that this will enable a much wider set of suitable phase retrieval approaches, depending on the application, similar to the state-of-the-art with SR based phase-contrast tomography.

Phase-contrast propagation imaging enables higher contrast for low absorbing tissues^5,20,21^ and increased spatial resolution, since it can invert the blurring by diffraction. If not taken into account, edge enhancement as the most prominent phase-contrast effect may look pretty in the (2d) projections, but results in disturbing edge-artifacts detrimental to the quality of the 3d data. This spoils automatic segmentation, visible for example in SR studies of cochleae carried out without phase retrieval^2,22^. To overcome this limitation, and primarily for comparison with our previous phase-contrast μ-CT work on tissues and organs, we use a fast Fourier transform-based phase-reconstruction procedure called the *Bronnikov Aided Correction (BAC)*^23^. In particular, we have shown that the obtained data, even at in-house laboratory sources, allows for automatic histogram-based segmentation between bone and soft tissue^1^, and that superior resolution can be reached^14^, compared to the Bronnikov (Fourier filter) approach^24^, or the popular inversion by Paganin’s formula^25^.

The approach is based on the transport of intensity equation (TIE), writing the measured intensity distribution *I*_*z*,*θ*_(*x*, *y*) at small propagation distances *z* behind an object with slowly varying absorption as^26–28^

1 $${I}_{z,\theta }(x,y)={I}_{z=0,\theta }(x,y)[1-\frac{z}{k}{\nabla }_{\perp }^{2}{\varphi }_{\theta }(x,y)]\mathrm{\ ,}$$

where *θ* denotes the projection angle, *k* the wavenumber, *ϕ*_*θ*_(*x*, *y*) the phase, and *I*_*z*=0, *θ*_(*x*, *y*) the intensity distribution in the exit plane of the object^1^. Next, using the modified Bronnikov algorithm (MBA)^24,29^, we can generate an approximation ${\tilde{\varphi }}_{\theta }(x,y)$ of the phase function, using a Fourier filter of the form *q(ξ,η*) = 1/(*ξ*^2^ + *η*^2^ + *α*), where *ξ* and *η* are the spatial frequencies and *α* is an absorption-dependent regularization parameter^1,30^. This is approximative, since the actual intensity distribution behind the object *I*_*z*=0, *θ*_(*x*, *y*) is unknown, and without regularization one would falsely attribute the entire signal to phase contrast. While at least partly quantitative, such approximative phase reconstructions are typically blurred^14,31^, and should therefore be further processed. To this end, the BAC uses a subsequent reconstruction step yielding the amplitude ${I}_{z=\mathrm{0,}\theta }(x,y)=\int \exp [-\mu (x,y,z)]{\rm{d}}L$ in the exit plane of the object

2 $${I}_{z\mathrm{=0,}\theta }(x,y)=\frac{{I}_{z,\theta }(x,y)}{1-\gamma {\nabla }_{\perp }^{2}{\tilde{\varphi }}_{\theta }(x,y)},$$

where *z*/*k* was replaced by the *α*-dependent regularization parameter *γ*. Using the two parameters *α* and *γ*, one can now reconstruct the projection with superior resolution, and an effective contrast mixing absorption and phase contributions. Note that this restriction of not separating phase and amplitude information is similar to single-material assumptions employed in several other phase retrieval schemes. With this non-iterative approach at hand, tomographic scans were recorded at a single distance.

## Results

**Figure 1:**
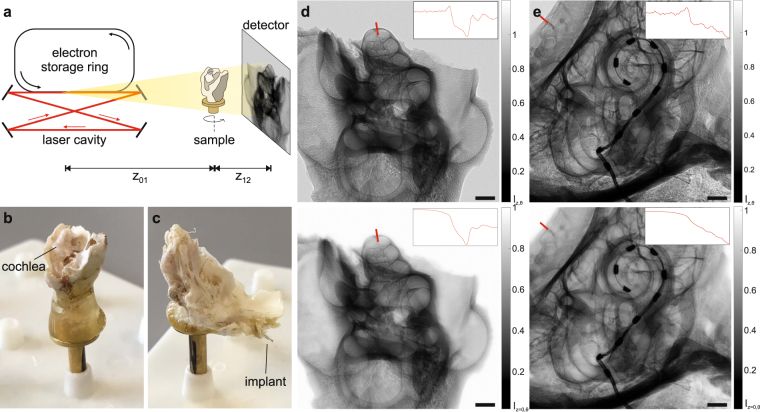
(**a**) Schematic of the experimental setup consisting of the Munich Compact Light Source (MuCLS) with an electron storage ring and a laser cavity, the sample on a motorized sample stage and a fiber-coupled sCMOS detector positioned at variable distance behind the sample. (**b,c**) Cochlea samples of guinea pig and marmoset on sample mount. Strong scattering of visible light impedes inspection of interior structures. (**d,e**) Recorded projections of the guinea pig cochlea (d,top) and the marmoset cochlea, the latter including the electrical CI (e, top), showing the edge enhancement emerging after wave propagation and the corresponding reconstructions using the *Bronnikov Aided Correction* (BAC) phase retrieval algorithm (bottom). The effect of the phase contrast in form of edge enhancement can also be observed in the profiles along an outer edge. By the BAC reconstruction, the overshoot of the edge profile is reversed and the original density profile is reconstructed, while preserving the sharpness and increasing the signal-to-noise ratio. Scale bars: 1 mm.

To evaluate the achievable data quality for samples at the small animal organ level, the cochlea of a guinea pig was imaged. To this end, the cochlea was carefully dissected from the skull, chemically fixed and afterwards glued to a sample holder (see Fig. 1(b)). Note, that this study was carried out as a pilot study in order to evaluate the achievable contrast with this new type of source. Therefore, no animals were sacrificed for this experiment but previously examined samples were used. As these were prepared weeks prior to this experiment, they were already dried out which led to the rupture of inner membranes such as the basilar or Reissner’s membrane. For the tomographic scan, phase-contrast images at 2500 projection angles distributed over 360° were recorded at an object-to-detector distance *z*_12_ = 1077.5 mm. Geometric magnification $M=({z}_{01}+{z}_{12})/{z}_{01}=1.29$ resulted in an effective propagation distance ${z}_{{\rm{eff}}}={z}_{01}\cdot {z}_{12}/({z}_{01}+{z}_{12})=844$ mm and an effective pixel size ${p}_{{\rm{eff}}}=p/M=5.05$ µm. The accumulation time per projection was 2 s, resulting in a total scan time of 2.5 hrs (including readout overhead, motor movement, empty images and darks). For an overview, all experimental parameters are listed in Table 1.

**Figure 2:**
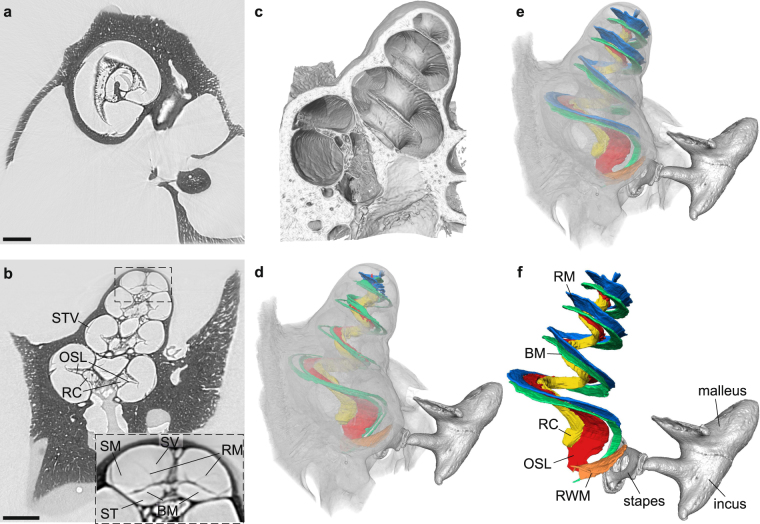
Reconstruction results for the guinea pig cochlea without implant. (**a,b**) Orthogonal virtual slices through the reconstructed 3d volume, showing the typical anatomical features of the cochlea in high detail without typical artifacts like beam hardening. In particular, the Rosenthal’s canal (RC), the osseous spiral lamina (OSL) and the stria vascularis (STV) can be recognized. In the inset, in which contrast was optimized for the soft tissue components, also the basilar membrane (BM) and the Reissner’s membrane (RM) are visible as well as the corresponding chambers separated by these membranes, the scala tympani (ST), scala media (SM) and the scala vestibuli (SV). (**c**) 3d rendering of part of the volume with a cut revealing the inner structure of the cochlea in high detail. (**d**) Segmentation of typical anatomical features of the cochlea together with a volume rendering displayed semi-transparently to put it in context. In blue (RM) and green (BM), the ruptured membranes due to drying are shown. (**e,f**) The same segmentation as in (d) but with the theoretical shape of the unruptured membranes derived from the position of typical landmarks in the volume. The segmentation includes the ossicles (malleus, incus and stapes), the round window membrane (RWM) as well as the OSL, RC, RM and BM. Scale bars: 1 mm.

Figure 2 shows the reconstruction results. Segmentation and analysis of the 3d data was performed using Avizo Fire 7 and Avizo 9 (FEI Visualization Science Group, Hillsboro, USA). Orthogonal slices through the reconstructed 3d volume are presented in (a) and (b), revealing anatomical features like the Rosenthal’s canal or to some extent even the thin basilar and Reissner’s membranes in high detail without typical artifacts such as beam hardening. For a better visualization of the 3d data, a volume rendering was created in which all background voxels are rendered fully transparent. For inspection of the inner structure of the cochlea, a virtual cut through the rendering shows the typical spiral-shaped cavity and corresponding features such as the osseous spiral lamina (Fig. 2(c)). Contrast and resolution are also high enough to identify and label specific features in the volume. The ossicles could be segmented using the built-in *magic wand* tool of Avizo which uses a region growing algorithm based on a user specified gray value range. The Rosenthal’s canal (yellow), the osseous spiral lamina (red) and the round window membrane (orange) could be labeled by marking the respective structures in a few slices, using the *brush* tool which only considers voxels in a limited grey value range, and subsequent interpolation. The thin membranes within the cochlea posed a challenge due to rupturing caused by drying over time. By simply segmenting those ruptured membranes, the 3d segmentation is heavily disturbed and it does not resemble the native structure of the cochlea (see Fig. 2(d)). However, by estimating the theoretical shape of the membranes based on specific landmarks in the 3d volume, the structure of the freshly prepared cochlea can be approximated (Fig. 2(e,f)). Further, by using freshly prepared cochleae, unbroken thin membranes can be visualized in a physiologically relevant state, as shown later in the manuscript for a tomography scan at a laboratory setup with a liquid metal jet anode (LMJ), which is described in more detail below.

In a next step, we have investigated the influence of metallic components in the sample by imaging the cochlea of a marmoset with an electrical implant of which the petrous part of the temporal bone was dissected and glued to a sample holder (see Fig. 1(c)). As in the case of the guinea pig cochlea, the sample was already prepared weeks before the experiment.

**Figure 3:**
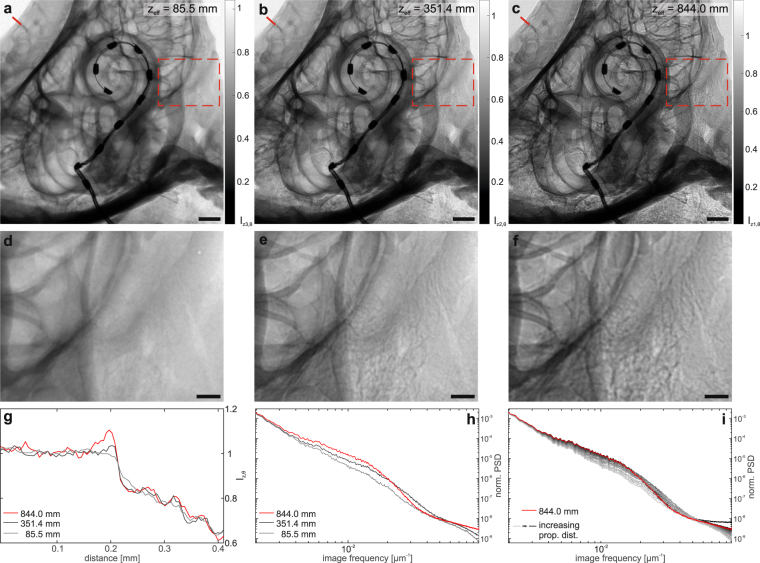
Effect of the propagation distance on contrast formation. (**a**–**c**) Projections recorded at the effective propagation distances *z*_eff_ = 85.5 mm (*z*_02_ = 3856.5 mm), *z*_eff_ = 351.4 mm (*z*_02_ = 4156.5 mm) and *z*_eff_ = 844.0 mm (*z*_02_ = 4856.5 mm), with corresponding zoom regions (dashed red rectangle) shown in (**d**–**f**). The zoom regions correspond to a size of 2.16 mm × 2.83 mm. The emergence of phase contrast with increasing *z*_eff_ is clearly evidenced and can be further evaluated by considering the profiles along an outer edge shown in (**g**). With increasing propagation distance the edge-enhancement effect gets stronger which results in a higher contrast. (**h**) The normalized angular averaged power spectral density of the projections shows the higher contrast for the projection at largest propagation distance for medium spatial frequencies, but also indicates the loss in resolution due to source blurring which can be recognized at the signal drop for higher spatial frequencies and the earlier transition to noise level. (**i**) Normalized angular averaged PSD for all recorded projections at *z*_02_∈[3856.5,4956.5] mm (in 50 mm steps), showing the evolution of contrast with increasing propagation distance (see also Suppl. Movie). Scale bars: 1 mm (**a**–**c**) and 300 μm (**d**–**f**).

Before turning to the results of the tomographic scan, the imaging properties of the MuCLS are further evaluated in 2d. To this end, a defocus scan is performed in which the source-to-object distance is kept constant at *z*_01_ = 3769 mm while the source-to-detector distance is increased from *z*_02_ = 3856.5 mm to *z*_02_ = 4956.5 mm in 50 mm steps. Three exemplary projections from this scan are shown in Fig. 3(a–c) with insets depicting a small region in higher detail (d–f). Due to the small propagation distance in (a) this projection corresponds to an (almost) pure absorption image of the sample. Note that the magnification changes with the source-to-detector distance, so all images are scaled to the smallest effective pixel size of 4.9 μm for comparison. Based on visual inspection, contrast seems enhanced with increasing propagation distance and already in these 2d projections details such as the sub-structure within the bony tissue surrounding the cochlea become visible which are not resolved in the absorption image (see especially the insets in (d–f) and also Suppl. Movie). This visual impression can be quantified by profiles along sample features like the outer edge of the cochlea (red line in the projections) which are shown in Fig. 3(g). While no edge-enhancement effect can be observed for the absorption image, it becomes stronger with increasing propagation distance resulting in higher contrast. As this behaviour is predicted by theory, this demonstrates that the coherence properties of the MuCLS setup are sufficient for propagation-based imaging. As a last step, the contrast transfer is evaluated by computing the power spectral densities (PSDs) of the recorded projections. In (h) the angular averaged PSD of the projections in (a–c) is shown. For comparison, all curves are normalized by the respective maximum value. The results indicate that for medium spatial frequencies $(\sim {10}^{-2}\frac{1}{\mu {\rm{m}}})$ a higher contrast can be achieved by a larger propagation distance while at the same time resolution is lost which can be recognized at the comparably large signal drop for higher spatial frequencies ($> 2\cdot {10}^{-2}\,\frac{1}{\mu {\rm{m}}}$) and the earlier transition to noise level. This tendency can also be observed when considering all images of the defocus series (Fig. 3(i)). The resolution drop can be explained by source blurring which increases with larger magnification^33^. Therefore, the detector distance is always a compromise between enhanced contrast due to phase-contrast effects and diminished resolution due to source blurring. It has to be chosen depending on the sample properties and goal of the study. In this case, we aimed for high contrast and therefore set the detector distance to *z*_02_ = 4856.5 mm. In the plots in (g - i) the corresponding curves are depicted in red.

**Figure 4:**
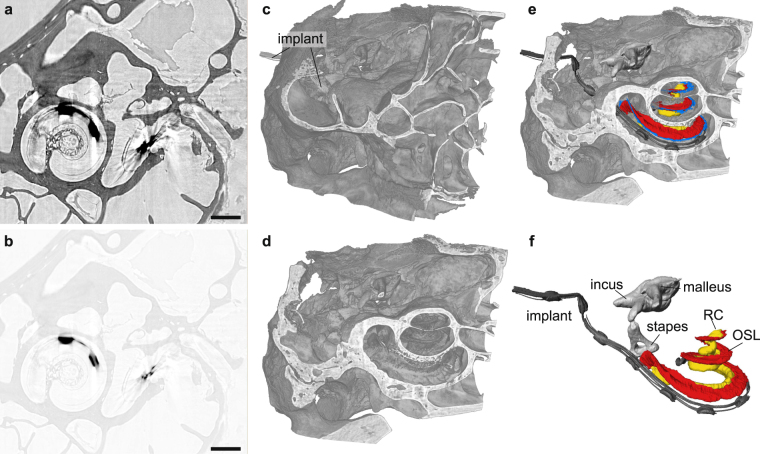
Visualization of the tomographic results obtained from a marmoset with an electrical cochlear implant. (**a,b**) Virtual slice through the object with two different contrast settings in order to optimize contrast for the bony structure and soft tissue (**a**) or the metallic implant (**b**). (**c,d**) Volume renderings of the sample showing the outer structure (**c**) and part of the inner structure. As the implant has significantly different gray values compared to the rest of the cochlea it could be excluded from the rendering in (**d**), leaving only the inner shape of the cochlea and the surrounding bones. (**e**) The same volume rendering as in (**c**) with typical structures segmented within the cochlea. As in the case of the guinea pig, the membranes (blue) are again ruptured due to drying. (**f**) The segmented structures show the ossicles, the Rosenthal’s canal (RC), the osseous spiral lamina (OSL) and the cochlear implant. Scale bars: 1 mm.

For the tomographic scan, the same parameters as before were chosen (see Table 1). In Fig. 1(e) an exemplary projection together with the respective reconstruction of the intensity distribution directly behind the object via the BAC algorithm is shown. When looking at the gray value range, it becomes evident that the metallic implant almost completely absorbs the incoming radiation which is a cause for the typical metal streak artifacts known from conventional clinical CT^34,35^. A slice through the reconstructed volume in which this effect can be observed is depicted in Fig. 4(a,b) with two different contrast settings in order to optimally represent the bony structure and soft tissue as well as the metallic components. In order to visualize the 3d information on the sample, again a volume rendering was created with the background voxels set to full transparency (Fig. 4(c)). The implant can be clearly recognized as it enters the bony structure from the left. In order to reveal the cochlea, which is fully enclosed by the surrounding structures, a virtual cut was made through the volume. Additionally, the metal implant was removed from the rendering based on the very different grey values of the metal components (Fig. 4(d)). As in the case of the guinea pig cochlea, also in this case typical features as the Rosenthal’s canal or the (ruptured) soft tissue structure of the Basilar membrane could be identified and labelled and are depicted in Fig. 4(e,f). Due to the artifacts, automatic segmentation was problematic, but nevertheless all features, including the ossicles, could be labelled by manually marking the respective structures in a few slices and subsequent interpolation. Note that also in this case the cochlea was already dried out, so that the membranes, depicted in blue, were again ruptured and therefore show no resemblance to the native structure of a freshly prepared cochlea. But as in the case of the guinea pig we expect the data quality to be high enough to also resolve the thin membranes in a freshly prepared hydrated cochlea, see below. Notwithstanding residual artifacts due to the metal implant and the structural alterations associated with the drying process, the results clearly show that the MuCLS setup can be used advantageously for non-destructive imaging of the electric implant and its exact positioning with respect to the different features of the cochlea which otherwise cannot be assessed.

Finally, we want to close the results with a brief comparison of the present results to those obtained with a liquid-metal-jet (LMJ) x-ray source^36–38^ with a Galinstan anode (68.5% Ga, 21.5% In and 10% Sn), similar to the results by Bartels *et al*.^1^. For a comparison at similar conditions, the same detector and the same effective pixel size were used. Due to the x-ray emission over the full angular range, the source-to-detector distance is chosen relatively small (*z*_02_=317.8 mm) in order to exploit a sufficient fraction of the flux for imaging. Note that this leads to a smaller object-to-detector distance compared to the MuCLS setup and therefore a decreased phase-contrast effect. A measure for this effect is the so-called Fresnel number which is defined as $F={p}^{2}/(z\cdot \lambda )$, with the pixel size *p*, propagation distance *z* and wavelength *λ*, or in the case of effective variables in the cone-beam geometry ${F}_{{\rm{eff}}}={p}_{{\rm{eff}}}^{2}/({z}_{{\rm{eff}}}\cdot \lambda )$. The smaller the Fresnel number the more pronounced the edge enhancement and therefore the higher the contrast (see Fig. 3, with increasing propagation distance the Fresnel number decreases). In this case, the Fresnel number is *F*_eff_ = 3.4558 which is roughly a factor of 5 larger than the Fresnel number of the MuCLS dataset (*F*_eff_ = 0.608). Notwithstanding that this leads to a decreased signal-to-noise ratio (SNR) in the LMJ datasets, a qualitative comparison is carried out in the following.

**Figure 5:**
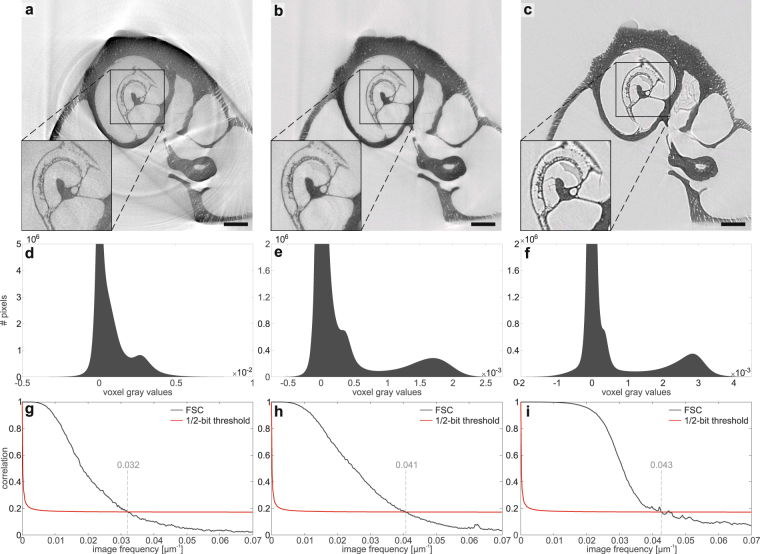
Comparison to the liquid-metal-jet setup. (**a**) Transverse slice through the reconstructed volume obtained at the LMJ source with the full spectrum (0–70 keV bremsspectrum, characteristic Ga-*K*_*α*_ line at 9.25 keV). The reconstruction is heavily degraded by beam hardening and ring artifacts due to the strong absorption by the bony structures. (**b**) Slice at the same position in the dataset acquired with a combination of a 25 μm thick nickel foil and a 35 μm thick silver foil for monochromatizing the radiation to the energy range of the In-K_*α*_ emission line at 24.2 keV. Artifacts are clearly reduced. (**c**) For comparison, a transverse slice at a similar position in the volume from the MuCLS dataset recorded at a photon energy of 25 keV. (**d–f**) Relevant part of the pixel gray value histograms of the reconstructed datasets. While the three components background, soft tissue and bony structures are not seperated in the unfiltered case, they can be very well distinguished in the other two cases. (**g**–**i**) Fourier Shell Correlations of the central 1000^3^ voxels of the reconstructed dataset as an estimate for the resolution. The unfiltered dataset shows the worst resolution due to the artifacts in the reconstruction but also a smaller SNR whereas the resolution of the other two datasets is higher, especially in the MuCLS dataset. Scale bars: 1 mm.

Here, two source configurations are considered: (i) a measurement using the whole spectrum of the source (Fig. 5(a)) and (ii) a measurement with an upstream filter consisting of a 25 μm thick nickel foil and a 35 μm thick silver foil which results in an x-ray beam with a main energy at the In-K_*α*_ emission line at 24.2 keV^12^ (Fig. 5(b)). The experimental parameters are again listed in Table 1. For comparison, the corresponding slice through the volume obtained at the MuCLS setup is shown in (c). Due to the broad spectrum of the LMJ with a main energy at 9.25 keV, the slice in (a) is heavily disturbed by beam-hardening artifacts such as streaks between high absorbing structures or cupping, which leads to a non-uniform density distribution in the homogeneous bone material. Additionally, the SNR is decreased due to the large absorption of photons which therefore cannot reach the detector (SNR ~9 in inner regions and ~16 in outer regions of bony structure). These effects can be minimized by using the filters: the slice in (b) shows almost no beam-hardening artifacts and an improved SNR (~30–35) since the main part of the spectrum is filtered out prior to recording the image, which enables longer exposure times before saturating the detector but also increases the total scan time. The results from the MuCLS setup show much less artifacts due to the high monochromaticity. Additionally, a large SNR of ~40 can be reached as the flux of 9 ⋅ 10^9^ ph/s allows for a good photon statistics in a relatively short exposure time and the long propagation distance leads to an increased contrast due to phase effects. Figure 5(d–f) shows the corresponding histograms of the 3d volumes reconstructed for the different setups. In the case of the full spectrum at the LMJ, the three components air, soft tissue and bone cannot be clearly distinguished. Contrarily, the histograms of the other two setups indicate a clear gray value separation and especially the result of the MuCLS setup shows very narrow peaks due to the high monochromaticity.

In a further step, we estimate the resolution of the different datasets by performing Fourier Shell Correlation (FSC)^39^ for the central 1000^3^ voxels of the reconstructed 3d volumes. To this end, two independent datasets are reconstructed, one from every second projection starting with the first and one from every second projection starting with the second. The resulting FSC curves are shown in (g-i, dark gray) along with the 1/2-bit threshold curve^39^ (red). The first crossover indicates the largest spatial frequency that can be resolved and yields a half-period resolution of 15.6 μm (g), 12.2 μm (h) and 11.6 μm (i), respectively. Thus, although the source size is a factor of four larger in the MuCLS setup, a slightly better resolution as in the LMJ case can be achieved in this configuration. Note that the LMJ resolution is also limited by the low SNR.

**Figure 6:**
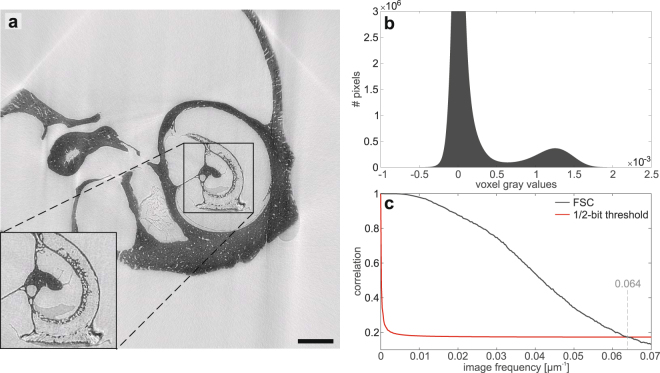
Results of the cochlea obtained with a more optimized setup for propagation-based imaging with a liquid-metal jet source. (**a**) Transversal slice through the reconstructed volumes at a similar position as in the measurements shown in Fig. 5 with an inset yielding the central part of the slice at higher detail. (**b**) Relevant part of the pixel gray value histogram of the reconstructed volume. The background peak as well as the peak of the bony structure are clearly visible whereas the soft tissue peak is barely visible in the form of a slight shoulder on the right side of the background peak. However, the slice clearly shows that soft tissue can be distinguished from background and bony structure. (**c**) Fourier Shell Correlation for the central 1000^3^ voxels of the reconstructed volume, showing a half-period resolution of 7.8 µm. Scalebar: 1 mm.

One possibility to increase the SNR in the LMJ setup is to increase the source-to-object distance as in the case of the Compact Light Source. However, this modality is not suitable due to the large cone of the liquid-metal-jet source and the associated loss in photon density. This requires long exposure times which can lead to sample changes due to degeneration of the sample or varying outer conditions and introduces artifacts in the reconstruction. Therefore, in order to increase the propagation distance we exchange the detector for a system with a larger pixel size. In this case the magnification needed to achieve an effective pixel size of 5.05 μm is larger so that a larger distance between the sample and the detector is necessary. This leads to a smaller Fresnel number and hence a larger influence of the phase information on the measured intensity image. This option is realized by replacing the sCMOS camera with a pixel size of 6.5 μm by a Flat Panel CMOS detector with a GdOS:Tb-scintillator screen (PerkinElmer, Waltham, USA) which has a pixel size of 74.8 µm. The source-to-object distance was set to *z*_01_ = 120 mm and the source-to-detector distance to *z*_02_ = 1783 mm, leading to a Fresnel number of 1.689 (in comparison to 3.4558 in the previously discussed geometry). For the tomographic measurement 2500 projections over 360° were recorded with an exposure time of 2.5 s each. In order to avoid beam hardening artifacts the combination of Ni and Ag foils was again used for filtering^12^. An exemplary slice at approximately the same position in the volume as in Fig. 5 is shown in Fig. 6 with the corresponding gray value histogram and Fourier Shell Correlation for the central 1000^3^ voxels of the reconstructed volume as an estimate for the resolution. Despite the same effective pixel size and source settings as in the previous geometry, resolution is increased by a factor of 1.5 to approximately 7.8 μm by changing to this optimized setup while exposure time was reduced by a factor of 4 due to the more efficient detector. In fact, resolution is even better than in the results obtained by the MuCLS setup. However, the SNR is slightly worse (~19–22) and the separation of gray values is not as clearly visible in the histrogram as before, although the slice suggests that the different tissue components can still be well recognized.

As only an already dried cochlea was imaged at the MuCLS setup, the question remains whether it can be used to resolve the thin membranes within the cochlea in a hydrated state. To estimate this, data from the same marmoset cochlea with implant shown above, acquired prior to the experiments at the MuCLS on the freshly prepared cochlea which was at the time of the measurement not yet dehydrated, can be considered. Until the measurement it was stored in PBS whereas during the measurement no further measures are taken to preserve humid conditions. Hence, fast acquisition times are needed to image the cochlea in the hydrated state as otherwise drying of the liquid-filled cavities and the attached soft tissue will occur. In order to increase contrast, it was measured in the optimized setup, using the Flat Panel CMOS detector and exploiting a larger propagation distance (source-to-sample distance: *z*_01_ = 115 mm, source-to-detector distance *z*_02_ = 1743 mm). Beam hardening artifacts were reduced by again using the filter combination of a 25 μm thick nickel foil and a 35 μm thick silver foil. The tomographic scan was carried out by acquiring 1000 projections over 183°, the extra 3° being necessary in order to avoid cone-beam artifacts. The counting time was 2 s per projection. For data analysis the scheme specified in the Materials and Methods section was used, with the regularization parameters *α* = 0.01 and *γ* = 0.053.

**Figure 7:**
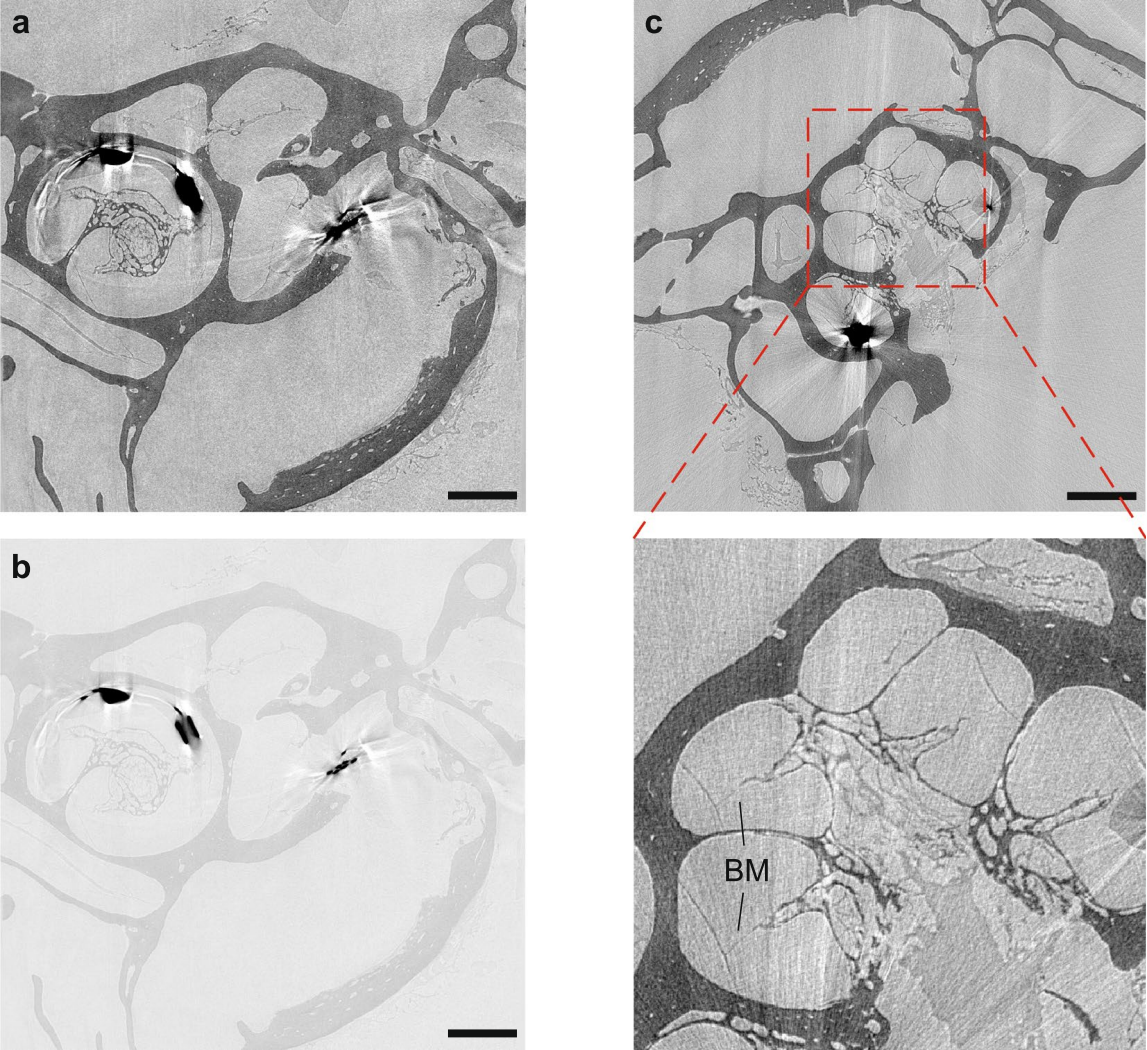
Virtual slices through the reconstructed volume from a marmoset with an electrical cochlear implant measured at the LMJ source. (**a,b**) Virtual slice at a similar position as for the MuCLS setup in Fig. 4. Again, two contrast settings are chosen in order to optimize it for the bony structures and soft tissue (**a**) and the metallic parts of the cochlear implant (**b**). (**c**) Orthogonal slice showing the unruptered basilar membrane which can be recognized despite the artifacts caused by the implant. This indicates that by imaging a freshly prepared cochlea it is possible to resolve the delicate soft tissue structures, as the thin membranes, without artifacts due to drying. Scalebars: 1 mm.

In Fig. 7 virtual slices through the reconstructed volume are shown. For a better comparison, a similar slice as in Fig. 4 is depicted, again optimized for contrast in the bone and soft tissue region (a) and for the cochlear implant (b). Also in this case, the metallic implant poses a challenge due to the low transmission which leads to strong artifacts in the reconstruction. However, the surrounding tissue is nevertheless well resolved an. In c it can be further recognized that even the thin basilar membrane is visible despite the artifacts caused by the electrical implant.

As the sample was in this case measured prior to the experiment at the MuCLS setup and was actually freshly prepared, the membrane is still intact. The previously presented results, especially those in Figs 5 and 6, suggest that both the MuCLS and the optimized LMJ setup lead to comparable reconstructions. Therefore, the result obtained for the hydrated cochlea at the LMJ setup makes us confident that also with the MuCLS imaging of the cochlea in its native state with intact soft tissue structures as the thin membranes should be possible.

The comparison between the MuCLS and the LMJ setups highlighted above (for identical pixel sizes) shows that the low photon energy of the current LMJ source impedes investigation of cochleae of larger animals, such as the marmoset cochlea studied here, and that only by monochromatizing the radiation to the In-*K*_*α*_ emission line high quality measurements can be performed. Using the same detector as in the MuCLS experiment, this comes at the cost of long exposure times which may lead to sample degradation during the measurements. Note, however, that a metal-jet source with mean energy of 24 keV has recently been developed^40^, which may further extend the potential of 3d cochlea imaging in this LMJ geometry. By changing the laboratory setup to a more optimized geometry by exchanging the detector and exploiting a larger propagation distance, similar exposure times and even a slighty better resolution compared to the MuCLS setup can be reached. However, this comes at the cost of a lower signal-to-noise ratio and a less clear separation of gray values which might impede automatic segmentation in some cases. Further, the dose required to obtain comparable image quality is higher for the LMJ than for MuCLS, see Table 1

**Table 1:** Experimental parameters for the tomographic scans.

	Fig. 2	Fig. 4	Fig. 5(a)	Fig. 5(b)	Fig. 6
Brightness $[\frac{{\rm{photons}}}{{\rm{s}}\cdot {{\rm{mm}}}^{2}\cdot {{\rm{mrad}}}^{2}}]$	3.2 × 10^10^	3.2 × 10^10^	1.3 × 10^11^	3.1 × 10^10^	3.1 × 10^10^
source spot (h × v) [m^2^]	38 × 45 (*σ*)	38 × 45 (*σ*)	10 × 10 (FWHM)	10 × 10 (FWHM)	10 × 10 (FWHM)
*z*_01_ [mm]	3769	3769	247	247	120
*z*_02_ [mm]	4856.5	4856.5	317.8	317.8	1783
effective pixel size [μm]	5.05	5.05	5.05	5.05	5.05
exp. time/proj. [s]	2	2	0.8	10	2.5
number of projections	2500	2500	2501	2501	2500
angular range [°]	360	360	360	360	360
BAC, *α*	0.005	0.005	0.05	0.03	0.02
BAC, *γ*	0.25	0.26	0.029	0.035	0.09
surface dose [Gy]	60	60	252	228	242
half period resolution [μm]	11.6	14.2^*^	15.6	12.2	7.8
SNR	40	29	9–16	30–35	19–22

Brightness instead of brilliance is chosen as a measure due to the large bandwidth of the bremsspectrum in the case of the laboratory source. Note, that this bandwidth is therefore not represented in the values specified here and that in the brightness for the MuCLS setup the considered photons are of similar wavelength whereas for the brightness of the laboratory source photons from the entire spectrum were considered. Dose values were calculated for a model protein^32^ (density *ρ* = 1.35 g/cm^2^) with weighted spectral contributions for the laboratory setup. Signal-to-noise ratios (SNRs) were determined between bone structure and air on an arbitrarily chosen virtual slice.

*This value is estimated via the edge steepness of an edge between bone and air. The given value is half of the FWHM of an error function fitted to the edge in order to get the half period resolution. As the reconstruction is dominated by the sharp artifacts due to the metal implant, the resolution via the FSC was estimated too high, as confirmed by visual inspection. Therefore it could not be used as a measure for resolution for the given dataset.

. While the brightness values of the two sources are comparable, the narrow photon energy distribution of MuCLS is more favorable for phase contrast.

## Discussion

The results demonstrate that the Compact Light Source is well suited for 3d imaging of structurally preserved soft tissue surrounded by bone, with a field of view large enough to cover an entire organ of small animals, using phase contrast based on free propagation in the direct contrast regime (TIE-regime). The strength of the CLS for objects with a size at the organ level is evidenced both in terms of the relatively short scan times and the obtained data quality especially with respect to signal-to-noise ratio, quantitative gray values and the minimization of artifacts such as beam hardening which are typical for conventional laboratory setups using the broad spectrum of the bremsstrahlung. In particular, phase retrieval yields an image quality high enough for semi-automatic histogram-based segmentation between bone and soft tissue. This enables fast and convenient data visualization as required by biomedical studies and demonstrated here for cochlea research. Note, that an exchange of the laser system subsequent to the experiments shown here now provides a flux that is even an approximate factor of 2 higher.

Further, for cochleae with electrical implants, the narrow bandwidth of MuCLS enables reconstructions with relatively few artifacts in the area of the bony structures or soft tissue, but strong metal streak artifacts due to the still relatively low photon energy. However, data quality is still sufficient to evaluate the position of the implant and the respective position of the nerve in the investigated cochleae. Additionally, the photon energy can in future be further increased by adjusting the electron energy, reducing the artifacts caused by the strongly absorbing implant. Contrarily, with the current anode material the photon energy of the LMJ source is limited by the characteristic In-*K*_*α*_ line at 24.2 keV, which is not sufficient to make the metal transparent.

We expect that propagation imaging using Compact Light Sources can be used for a broad range of biomedical imaging applications in which 3d data with moderate resolution and comparatively large FOV has to be obtained for soft tissue. In particular, scan times and availability should be compatible with biomedical studies requiring a high throughput to achieve statistically relevant results.

### Sample preparation

Cochleae were obtained from sacrificed adult Hartley guinea pigs (Cavia porcellus) and common marmosets (Callithrix jacchus). Insertion of a ten contact electrical cochlear implant was performed on the fresh marmoset skull. After preparation the samples were fixated in 4% paraformaldehyde for 1h. All experiments conformed to the local and national guidelines for the care and use of laboratory animals in research and were approved by the local authorities of the State of Lower Saxony (LAVES).

### Munich Compact Light Source

The Munich Compact Light Source (see Fig. 1(a)) consists of an electron storage ring of 4.6 μm circumference and a passive high-finesse laser enhancement cavity, which is driven by a Nd:YAG laser (wavelength: 1064 nm, pulse length: 25 ps, repetition rate: 65 MHz, ~14 W output power after amplification) and stored at the time of the experiment at about 130 kW. The electrons are accelerated by a linear accelerator to the desired energy in the range between 25 MeV and 45 MeV. At the interaction point of electrons and laser photons, x-rays are generated by inverse Compton scattering of the laser light at the electrons. The energy of the generated x-rays depends on the electron energy, the laser wavelength and the backscattering angle relative to the electron beam. Therefore, the spectrum of the x-ray beam can be tuned by placing a ring aperture downstream of the interaction point which limits the divergence of the beam to 4 mrad and hence narrows the spectral bandwidth to 3–5% (FWHM). In this experiment we chose an electron energy of 37.5 MeV, resulting in an almost monochromatic x-ray beam with a peak energy at *E*_*ph*_ = 25 keV and photon flux of 9 ⋅ 10^9^ ph/s. The source size was approximately 38 μm (*σ*_h_) × 45 μm (*σ*_v_) and the source-to-object distance was fix at *z*_01_ = 3769 mm, while the source-to-detector distance could be varied by moving the detector on its motorized stage *z*_02_∈[3856.5, 4956.5] mm, leading to object-to-detector distances of *z*_12_∈[87.5, 1187.5] mm. For the detection of x-rays, a fiber-coupled scintillator-based sCMOS camera (2048 × 2048 pixels, Photonic Science, Sussex, UK) with a custom scintillator (15 μm thick GdOS:Tb) and pixel size *p* = 6.5 μm was used.

### Liquid-metal jet setup

A detailed description of the liquid-metal jet setup can be found in previous works^1,13^. It consists of a liquid-metal jet source (anode: 68.5% Ga, 21.5% In and 10% Sn) with a characteristic photon energy of 9.25 keV (Ga-K_*α*_ emission line), a motorized sample stage as well as a detection system placed some distance behind the sample. The source was operated at a tube voltage of 70 kV with 100 W e-beam power at a spot size of 10 × 40 μm^2^ (FWHM), resulting in a projected source size of about FWHM_src_ = 10 μm. The source-to-sample distance can be varied via a translational axis while the source-to-detector distance is adjusted by hand. As a detection system either the same fiber-coupled scintillator-based sCMOS camera as in the MuCLS setup or a Flat Panel CMOS detector with a 150 μm GdOS:Tb-scintillator screen (PerkinElmer, Waltham, USA) was used.

### Data analysis

The intensity distributions *I*_*z*__=__0,*θ*_(*x*, *y*) were reconstructed for *θ* ∈[0,360]° from the empty-beam corrected projections using the BAC algorithm (see Fig. 1(d) and (e) for an exemplary projection). The parameters *α* and *γ* for each experiment, found by visual inspection, are listed in Table 1. Prior to tomographic reconstruction of the datasets, a simple ring removal algorithm was applied^41^. Due to the low divergence of the MuCLS setup, subsequent 3d reconstruction of the effective attenuation coefficients *μ*(*x*, *y*, *z*) was in this case performed via the built-in iradon function in Matlab (The MathWorks, Natick, USA) with a standard Ram-Lak filter whereas for the LMJ setup the cone-beam reconstruction algorithm of the ASTRA toolbox^42,43^ was used.

## Electronic supplementary material

Supplementary Information
